# Improving Greater Caribbean manatee vocalization detection across habitats using neural networks

**DOI:** 10.1371/journal.pone.0341561

**Published:** 2026-02-13

**Authors:** Eric A. Ramos, Amit Galor, Michael Faran, Michael M Mishelashvili, Nataly Castelblanco-Martinez, Marisa Tellez, Beth Brady

**Affiliations:** 1 Mote Marine Laboratory, Sarasota, Florida, United States of America; 2 Fundación Internacional para la Naturaleza y la Sustentabilidad, Chetumal, Quintana Roo, Mexico; 3 Deep Voice, Tel Aviv, Israel; 4 El Colegio de la Frontera Sur, Department of Systematic and Aquatic Ecology, Laboratory of Aquatic Mammals, Chetumal, Quintana Roo, Mexico; 5 Crocodile Research Coalition, Stann Creek, Belize; 6 Save the Manatee Club, Longwood, Florida; Universidad Miguel Hernandez de Elche, SPAIN

## Abstract

The detection and classification of Greater Caribbean manatee vocalizations (*Trichechus manatus manatus*) present unique challenges due to the complexities of underwater acoustic environments. This study explores the application of neural networks for improving the identification and classification of Greater Caribbean manatee vocalizations, which can provide valuable insights into their behavior and aid in conservation efforts. Utilizing a large dataset of underwater recordings, we trained a known CNN architecture without domain-relevant pretraining to identify and classify Greater Caribbean manatee calls. Our approach combined advanced signal processing techniques such as filtering and normalization with deep learning algorithms to account for the dynamic and noisy conditions of marine environments, employing data augmentation and feature extraction strategies to focus on relevant and informative sound characteristics. The neural network demonstrated promising results, with an overall F1 score of 95.6% on the Wildtracks test dataset, and an F1 score of 64.4% on the Placencia dataset after fine-tuning on less than 10 seconds of vocalizations. This highlights the ability of the model to generalize to novel datasets collected in different regions with vastly different noise profiles. Although there is room for improvement in terms of generalization, these findings represent an advancement in the automated detection and classification of Greater Caribbean manatee vocalizations. This could potentially lead to more effective monitoring of their populations and contribute to the development of improved conservation strategies.

## Introduction

In recent years, there has been a growing interest in the application of machine learning and neural networks for the detection and classification of bioacoustic signals [1,2]. Neural networks excel at processing large volumes of data, enabling the analysis of long-term, continuous recordings across diverse environments [3]. These methods have been successfully applied to various species, including primates [4], bats [5], and birds [6]. Deep learning architectures, such as convolutional neural networks (CNNs) and recurrent neural networks (RNNs), have demonstrated their ability to effectively learn complex patterns and extract relevant features from raw data, resulting in significant advancements in various bioacoustic applications [1,7]. For example, CNNs have been used to classify bird vocalizations to the species level based on spectrograms with high accuracy, automating the process of bird monitoring and identification in ecological research [6,8]. Similarly, CNNs have been employed to identify and classify cetacean vocalizations, which can enable valuable insights into the behavior and distribution of these marine mammals [9].

Furthermore, the application of data augmentation techniques can increase the robustness and generalization capabilities of neural networks, allowing them to perform well in real-world scenarios [10]. For instance, augmentations such as time-stretching, time-masking, and the addition of background noise can simulate diverse acoustic environments and improve model adaptability [11,12]. These methods enhance the neural network’s ability to detect vocalizations across various conditions, such as varying signal-to-noise ratios and environmental distortions [13].

Despite the potential of neural networks in the field of bioacoustics, relatively few studies have focused on the application of these techniques to the detection and classification of manatee vocalizations, particularly those of the Greater Caribbean manatee (*Trichechus manatus manatus*). Previous research on manatee vocalizations have primarily relied on manual analysis or traditional signal processing methods [14], which can be time-consuming and often lack the precision required for accurate detection and classification [15]. Consequently, there is a need to explore novel approaches that can efficiently process large volumes of acoustic data while providing accurate and reliable results.

Manatees, belonging to the family Trichechidae, are large aquatic mammals found in shallow coastal waters, estuaries, and rivers. They play a vital role in maintaining the balance of the marine ecosystem and serve as an indicator species for the health of these environments [16]. The Greater Caribbean manatee, a subspecies of the American manatee, is found throughout the Gulf of Mexico and the Caribbean [17]. Despite their ecological importance, Greater Caribbean manatees are currently listed as endangered, with their populations facing numerous threats, including habitat loss, boat strikes, and entanglement in fishing gear [18]. The development of effective monitoring techniques is crucial for understanding their distribution and mitigating these threats. Recently, a study of African manatees determined that acoustics was the best method to document manatee occurrence [19].

Manatee vocalizations are essential for communication and maintaining social bonds within their populations [20,21]. Their vocalizations are characterized by a series of short, high-pitched calls that cover a frequency range of approximately 2–6 kHz, with each call lasting around 300–800 milliseconds [22,23]. Manatees primarily produce three call types that are used while traveling, during play, in stressful situations, and in interactions between cow-calf pairs [21,24]. This vocal activity makes them excellent candidates for passive acoustic monitoring which can be used to record manatee presence to aide conservation efforts by identifying critical habitats and informing effective conservation strategies [25].

In this study, we aim to investigate the potential of CNNs in detecting Greater Caribbean manatee vocalizations. We built a pipeline and trained a neural network architecture specifically for this task, based on the Soundbay platform [26] combining advanced signal processing techniques (e.g., spectrogram optimizations, different normalization techniques, filtering) with deep learning algorithms to address the challenges associated with the underwater acoustic environment. By employing data augmentation and feature extraction strategies, our model is designed to focus on the most relevant and informative characteristics of Greater Caribbean manatee vocalizations. We evaluated the performance of our model using a large dataset of underwater recordings, assessing its detection accuracy and comparing it to other methods. Additionally, in order to test the generalization capabilities of the model, we used an independent test dataset which was not seen by the model during training. With this dataset we tested the model’s ability to detect vocalizations in recordings from a different domain in terms of geography, environment and sound characteristics.

## Methods

### Data collection

**Fig 1:**
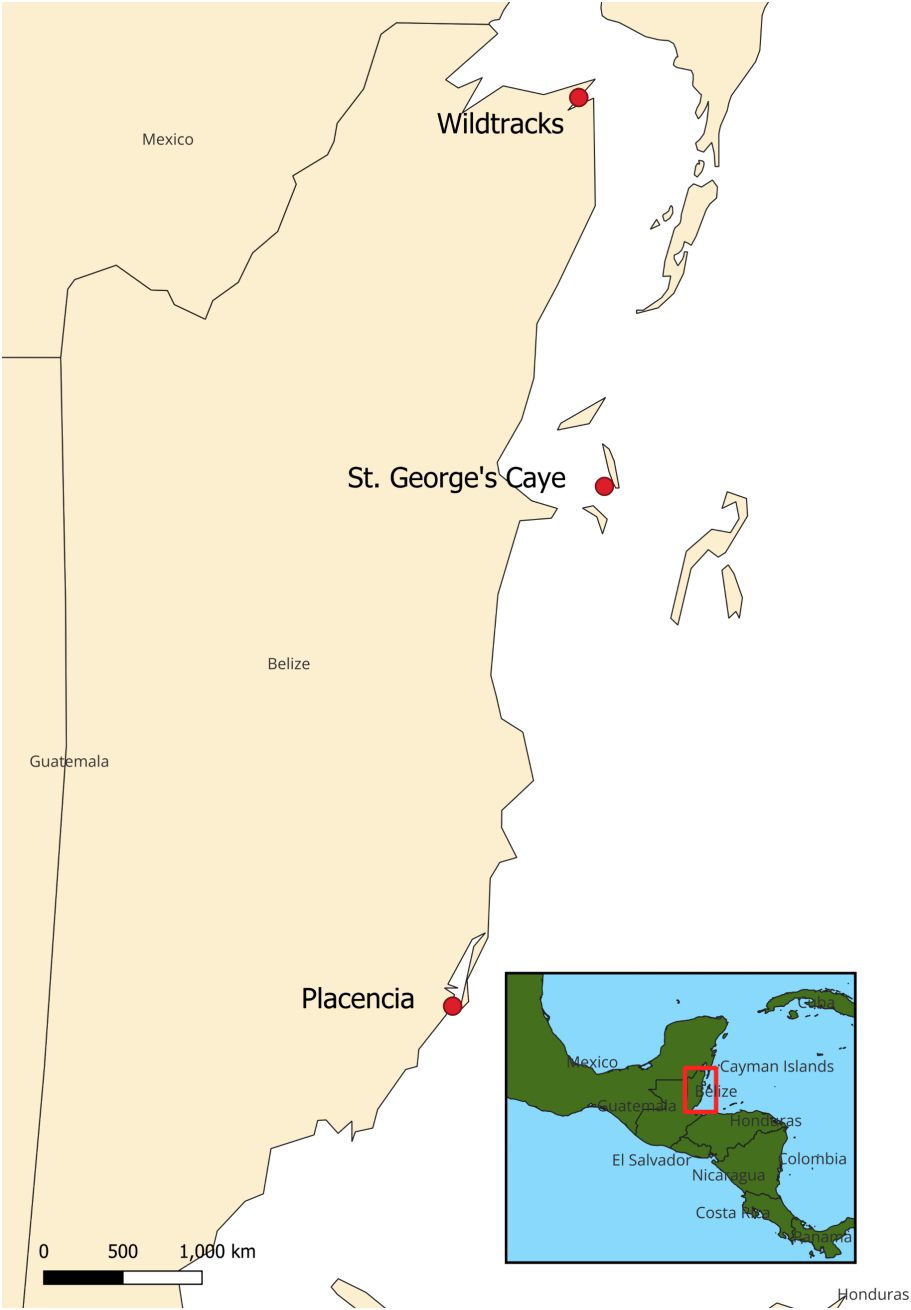
Map showing the locations of acoustic recorders in Belize used to collect manatee vocalizations for training models. The basemap and inset was created using the free and open source qGIS3 bundled world layer software version 3.40.4 (URL: https://qgis.org/).The base map was made using the Esri Gray Light layer (URL: https://www.arcgis.com/home/item.html?id=33ea4550c8144e66847d902e4766c2f7) and is licensed as open access under the Creative Commons 4.0 license.

Vocalizations of wild manatees were collected in three locations along the Caribbean Sea coast of Belize: Wildtracks, a captive rehabilitation facility in northern Belize, Placencia Lagoon in the Stann Creek District in southern Belize, and St. George’s Caye near the barrier reef east of Belize City (Fig 1). Wildtracks (www.wildtracksusa.org/) is a wildlife rehabilitation center responsible for the rehabilitation and release of wild orphaned and injured manatees. Recordings were obtained from ten captive individuals from December 8, 2021- February 27, 2022. St. George’s Caye (SGC), is a small crescent-shaped island located 9.5 km east of mainland Belize near the Belize Barrier Reef. Recordings were made July 8–10, 2017 and July 13–16, 2017 [27,28]. The caye is surrounded by expansive seagrass flats, sand patches, and deep channels and holes, and the area is regularly inhabited by manatees of all ages and sex classes [29]. Placencia Lagoon is a semi-enclosed, shallow, coastal estuarine system that is considered part of the Southern Belize Reef System. Placencia Lagoon is one of Belize’s key manatee habitats, primarily due to the presence of seagrass beds of *Halophila baillonii,* a preferred food for the species [30]. Vocalizations were recorded in Placencia Lagoon between January 27, 2021- February 28, 2022. Research was approved by the Belize Fisheries Department.

Acoustic recordings from Wildtracks were made with a SQ26–08 hydrophone (Cetacean Research Technology; sensitivity: −169 dB re 1 V/µPa) connected to a TASCAM DR-05 recorder which sampled at 96 kHz and 16 bits [31]. The hydrophone was placed approximately 1 m into the water column, housed within a custom-built PVC cage lined with nylon netting. The housing was needed to protect the hydrophone from the curiosity of the young manatees. All acoustic recordings of wild manatees were made with a calibrated SoundTrap 300 HF (Ocean Instruments, New Zealand; sensitivity: −188.4 dB re 1 V/μPa) that sampled continuously at a at 288 kHz in 16-bit (flat frequency response: 0.02–150 kHz [62 dB]) with the preamplifier gain on. The SoundTrap was anchored by rope to the seafloor with a cinderblock and suspended in the water column at a depth of 1 m above the seafloor in water 1.5 m deep. In St. George’s Caye the device was placed at the edge of a seagrass bed in a human-dredged channel, near a large hole often used by manatees as a resting hole. In Placencia Lagoon, the recorder was deployed in multiple areas in close proximity to one another around the northern section of the lagoon in muddy and seagrass substrates. All methods were performed in accordance with relevant guidelines and regulations as suggested in the ARRIVE guidelines.

### Data processing

**Fig 2:**
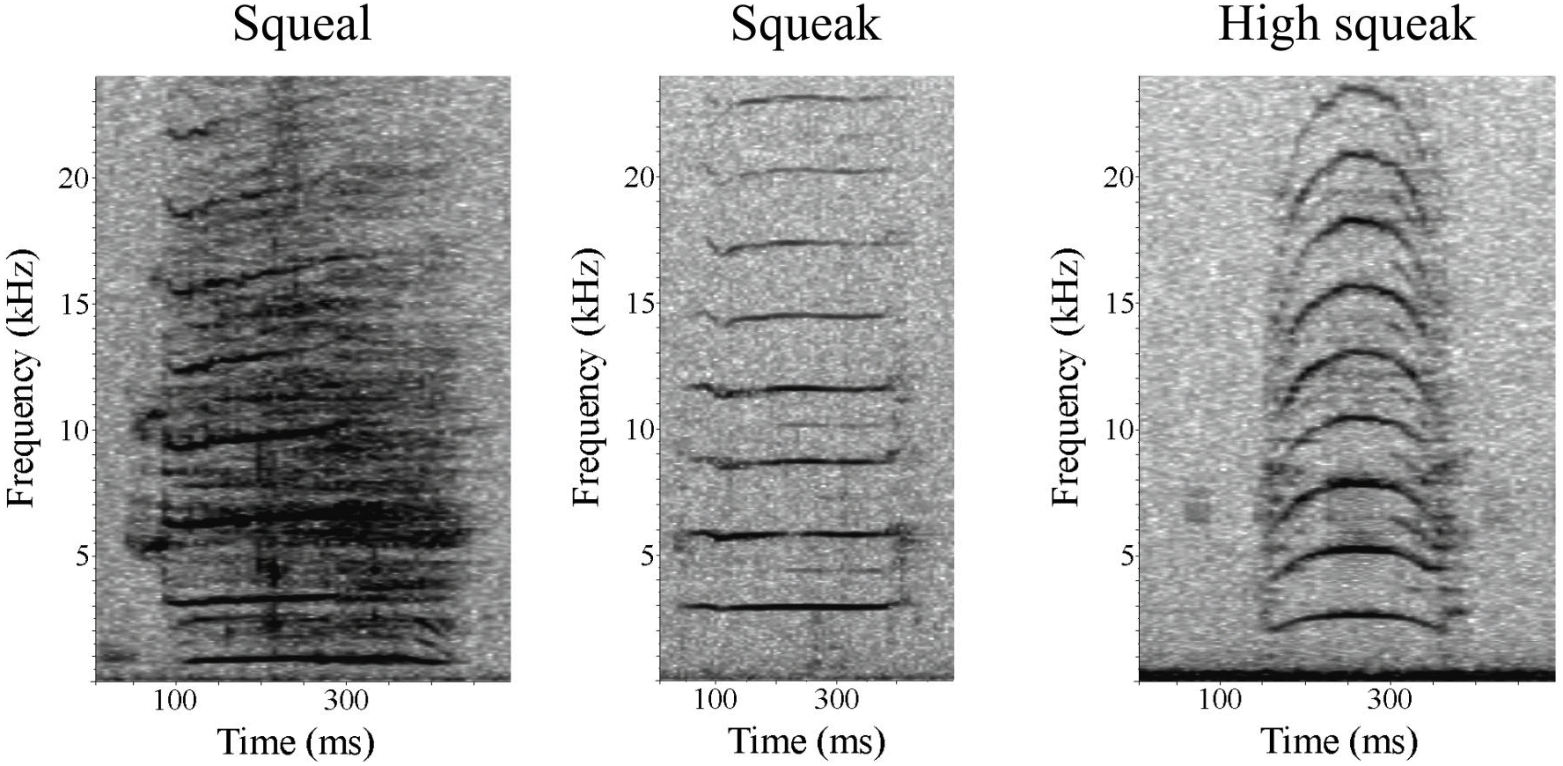
Spectrograms of common Greater Caribbean manatee vocalizations which include squeaks, squeals, and high squeaks. Spectrogram parameters: Hamming window, DFT: 1024.

To identify all manatee signals, Raven 1.6 was used to manually review all sound recordings (sample rate: 288 kHz/96KHz; DFT size: 2048 samples; Hann window; overlap: 90%; time resolution: 10.7 ms). Higher sample rate recordings were downsampled to 96 kHz before viewing. The selection feature was used to draw a box around each manatee sound (Fig 2). Manatee calls were manually detected by seven trained observers. Detected calls from the observers were then verified by two researchers skilled in identifying manatee vocalizations [23,28]. The annotated selection table for each wav file was saved and used to verify the accuracy of the detector.

The dataset from St. Georges’ Caye included ten wav files recorded on July 8–10, 2017, twelve wav files recorded on July 13–16, 2017, and their corresponding selection tables, all of which were used for analysis. Each wav file was 30 minutes long, with each call selected in the file having an annotated start and end time. Out of approximately 23 hours of recordings, a total of 2,066 annotated calls (“positive” samples) were identified, totaling 864.12 seconds.

The dataset from Wildtracks was annotated in the same method as previously described. From the 4 hours of recordings, 1,289 annotated calls were used for training, totaling 291.76 seconds of vocalizations. The Wildtracks test set included 316 vocalizations, amounting to 76.98 seconds. The Placencia generalization-test included 15 hours of annotated recordings, including 661 vocalizations amounting to 122.1 seconds

All the annotation files of the recordings marked for training-validation (St. George’s Caye and most of Wildtracks) were combined into a CSV file for each dataset, with all calls labelled as positive and background noise labelled as negative. Background noise samples were automatically extracted from the time intervals between the end of one annotated call and the beginning of the next. This process ensured that non-call sections of the recordings are included in the dataset as part of the negative class. The CSV was split into a training set and a validation set, as is standard in machine learning training. We chose an 80%/20% split for training and validation, respectively. This ratio was selected to ensure a sufficiently large validation set while maximizing the amount of data available for training. In the Wildtracks domain adaptation we chose a 20%/80% split to minimize overfitting and retain general features from the original training. The Placencia and St. George’s Caye data was downsampled to 96 kHz for efficiency, since most information from manatee vocalizations does not exceed 43 KHz [32]. The data were segmented into 0.2 sec intervals as this is similar to the average duration of manatee vocalizations [20,23].

Each 0.2s waveform slice (96 kHz; 19,200 samples) was transformed into a linear‐frequency magnitude spectrogram with torchaudio’s Spectrogram transform (fft = 1024, hop length = 256, Hann window). The spectrum was then converted to decibels (with torchaudio’s AmplitudeToDB function) and finally peak-normalized to the [0,1] range. This resulted in an input image size of 513 × 75 pixels (Frequency × Time), with a frequency resolution of 93.75 Hz and a time resolution of 2.67 ms. The same spectrogram pipeline was applied to training, validation, and test audio. Data augmentation techniques were used to increase the sample size and enhance the model’s generalization ability [33]. During training we applied a stochastic, on-the-fly augmentation pipeline implemented with Audiomentations v0.26 [34]. Each recording segment passed through an augmentation pipeline executed with overall probability of 0.9. When triggered, the following transforms were applied, each with the probability indicated in parentheses: time stretching (p = 0.5, rate 0.9–1.1), time masking (p = 0.5, hiding 5–20% of the window), and band-stop frequency masking (p = 0.5, center frequency sampled uniformly between 0 and the data’s maximum frequency (43kHz), bandwidth 5–20% of that center). No augmentations were applied to validation or test data. The exact YAML configuration is provided in the Soundbay repository [26]

### Model training

We adopted the ResNet-18 CNN architecture as implemented in *torchvision* and initialized it with ImageNet weights. The 18 convolutional layers are arranged in four residual stages (2 × [64, 128, 256, 512] filters). We replaced the original fully connected head with a 256-unit layer (ReLU + 0.5 dropout) followed by a 2-unit soft-max classifier, yielding the “ResNet18-2D” used throughout this study. The model was trained for 100 epochs with the Adam optimizer [35] (lr = 1 × 10 ⁻ ^3^, default betas = 0.9/0.999) with a scheduled learning rate decay after each epoch (γ = 0.995). Cross-entropy loss was minimized in all runs, and no early-stopping criterion was applied. The training configuration included treating “partial positives” (segments containing both a part of a call and some background noise time) as positive samples.

We conducted three sets of experiments to evaluate the performance and generalizability of our ResNet model architecture for manatee vocalization detection (Table 1).

**Table 1:** Overview of dataset usage and experimental objectives across training, validation, and test splits.

Experiment	Training data	Validation data	Test data	Purpose
1 – ResNet vs GoogLeNet	Wildtracks (80%)	Wildtracks (20%)	Wildtracks 3-day hold-out (test-W)	Architecture comparison
2 (Stage 1) – Base Training	St George’s Caye (80%)	St George’s Caye (20%)	–	Learn core features
2 (Stage 2) – Domain Adaptation	Wildtracks (20%)	Wildtracks (80%)	Wildtracks 3-day hold-out (test-W)	Low-resource domain adaptation
3 – Placencia transfer	- (zero-shot on model 2) or Placencia (3%/5%/10%/15%)	Placencia (2%)	Placencia remainder (test-P)	Cross-environment generalization

### Experiment 1 (Architecture comparison) – ResNet vs. GoogLeNet

To compare our ResNet-based method with alternate approaches [36], we trained both ResNet18-2D and GoogLeNet from ImageNet weights only on Wildtracks (with the same 80%−20% train-validation split, and “test-W” of 3 separate days), with both SGD and Adam optimizers. We used a similar preprocessing and training pipeline, except that the spectrograms for the GoogLeNet were converted to RGB images as in the cited work [30]. The models were trained for 30 epochs. This experiment provided a direct comparison of the detection capabilities of the ResNet and GoogLeNet models with the different optimizers and validated our choice of the ResNet architecture with the Adam optimizer.

### Experiment 2 (Base model + domain adaptation) ResNet model – Belize Dataset Split (St. George’s Caye and Wildtracks)

To evaluate the robustness of our selected architecture, this experiment assessed detection performance on a primary dataset and measured the efficiency of domain adaptation between two sites in Belize. There were two stages to this experiment. Stage 1 was to train ResNet18-2D on the St George’s Caye corpus (80% train, 20% validation). Stage 2 was to fine-tune that model on a Wildtracks subset (20% train, 80% validation) and evaluate on an unseen three-day Wildtracks hold-out set (test-W). This gauges how much low-resource in-domain data is needed to adapt the model within Belize. We used a 20% train/ 80% validation split for fine-tuning to limit overfitting on the small Wildtracks subset and help preserve generalizable features learned from St. George’s Caye. This configuration yielded better performance on the unseen Wildtracks test set and improved generalization overall.

### Experiment 3 (Cross-site generalization) – Placencia Dataset

**Fig 3:**
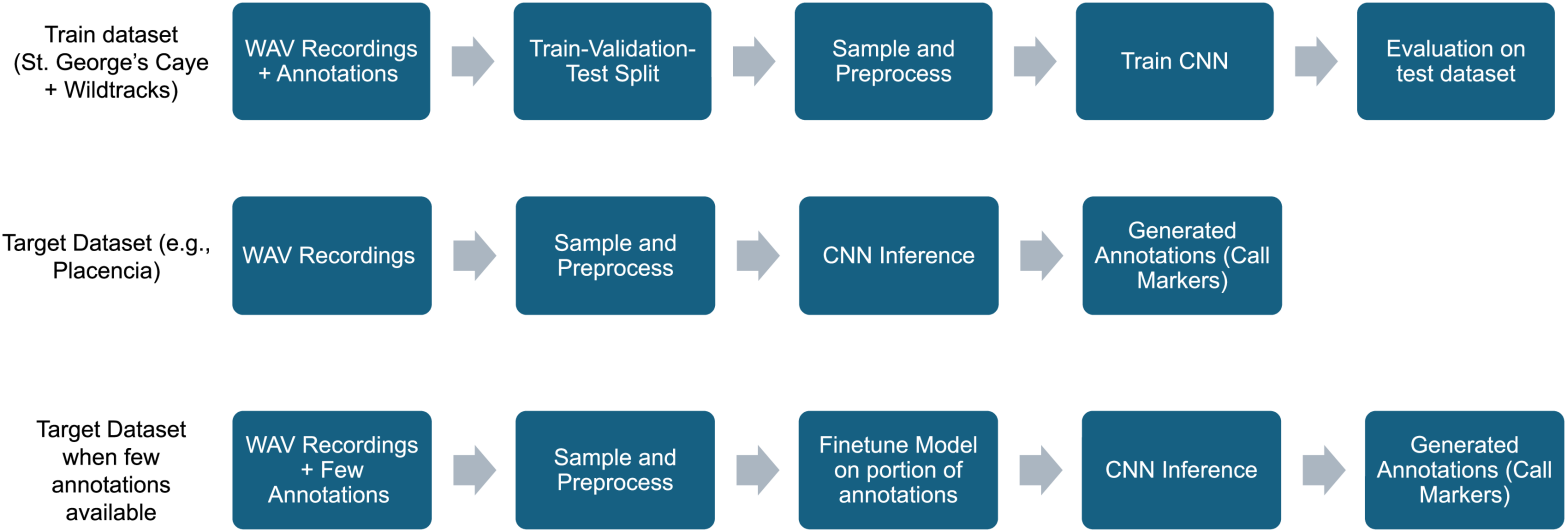
Workflow for applying a pre-trained model to a target dataset for call detection, with two approaches for the generalization challenge: direct inference or fine-tuning with limited annotations.

The last experiment demonstrates our structured approach for manatee vocalization detection in the wild (Fig 3). We begin by training a CNN on an annotated dataset. The trained model is then used for inference on a test dataset. Fine-tuning is performed using a small subset of available annotations before running inference again. This workflow allows us to maximize model generalization while minimizing the annotation effort for a new dataset.

To assess the generalization capability of the ResNet model (from Experiment 2), we conducted inference on the Placencia dataset, which was collected in a different acoustic environment with distinct sound profiles. The Placencia dataset was collected in an acoustically challenging environment than the training data, with higher background noise levels. This full Placencia corpus consisted of 185 recordings (~555h). For quantitative evaluation, five recordings (14h) were manually annotated post hoc for evaluation (“test-P”) and 661 vocalizations were found, i.e., ≈ 44 calls h ⁻ ¹. The remaining ~ 540h are un-labelled.

To evaluate the model’s generalization capability, we first conducted a blind test, running inference on the dataset without any fine-tuning. Additionally, we performed fine-tuning experiments on small annotated subsets (3%, 5%, 10%, and 15%, corresponding to approximately 1 second to 16 seconds of vocalizations) to explore the impact of limited annotation on model performance. Fine-tuning was carried out by continuing back-propagation on the entire ResNet18-2D checkpoint from Experiment 2, using the same spectrogram pipeline, augmentation scheme, and optimiser settings (Adam, learning rate = 1 × 10 ⁻ ^3^, γ = 0.995, batch size = 32), for only 3 epochs. The only variable was the proportion of annotated Placencia recordings provided for training (3%, 5%, 10%, 15%). In order to show the benefit of training first on a different domain, we also compared the results to models that were only trained on the small subsets of Placencia annotations without pretraining on previous manatees datasets.

### Correlation of temporal call density

Detection metrics such as F1 or precision penalize any false-positive equally, yet in ecological practice a detector is still helpful if it preserves the temporal pattern of vocal activity. We therefore created a graph of calls-per-hour and calls-per-minute to help researchers find peaks of bioacoustic activity in a vast recording corpus. We then computed both Pearson’s r (linear agreement) and Spearman ρ (rank agreement) correlation coefficients. We evaluated both the pre-trained and fine-tuned models that started from the Experiment 2 checkpoint and were fine-tuned on 3%−15% of Placencia data, as well as the no-pre-training models trained directly on the same 3%−15% subsets. All correlation coefficients were calculated on the fixed Placencia test set “test-P”.

### Evaluation metrics

Segment-level confusion matrix: Each 0.2s spectrogram slice is labelled positive if any portion of an annotated call overlaps the slice and negative otherwise. A slice predicted positive is a true positive (TP) when the ground-truth label is also positive; otherwise it is a false positive (FP). Conversely, a ground-truth positive slice missed by the model is a false negative (FN), and a correctly rejected slice is a true negative (TN).

Accuracy is therefore:


$$Accuracy=\frac{TP+TN}{TP+FP+FN+TN}$$

I.e. the proportion of segments classified correctly

Precision and Recall characterise the positive class (vocalizations):


$$Precision=\frac{TP}{TP+FP},Recall\;=\frac{TP}{TP+FN}$$

Their harmonic mean is the call F1 score:


$$F\text{1}\;=\;\frac{2\cdot Precision\cdot Recall}{Precision\;+\;Recall}$$

AUC-PR (area under the precision-recall curve) is obtained by sweeping the detection threshold over the model’s output scores. All metrics are computed on the fixed validation or test splits described in the data processing section.

## Results

### Experiment 1 Comparative Results – ResNet vs. GoogLeNet

In this experiment, both model architectures with the two common optimizers were trained on the 2022 Wildtracks train set with the same train parameters, and tested on the Wildtracks test set. The comparative analysis between ResNet and GoogLeNet models and between the SGD and Adam optimizers revealed significant differences in performance (Table 2)

**Table 2:** Comparative results of F1 score, precision, recall, accuracy and AUC-PR between GoogLeNet and ResNet18 with SGD/Adam optimizers.

Model+Optimizer	Call F1 score	Precision	Recall	Accuracy	AUC-PR
GoogLeNet + SGD	78.6%	71.1%	87.8%	75.7%	83.9%
GoogLeNet+Adam	68.1%	83.9%	57.3%	98.0%	86.9%
ResNet18 + Adam	80.5%	91.0%	72.2%	98.7%	94.5%
ResNet18 + SGD	5.9%	15.4%	3.6%	95.6%	63.2%

### Experiment 2: Results – Belize Dataset Split (St. George’s Caye and Wildtracks)

The ResNet model achieved highly accurate detection performance on the 2022 Wildtracks test set, after training on the 2017 St. George’s Caye and 2022 Wildtracks train set. Table 3 displays the confusion matrix, which summarizes the model’s performance by comparing its predictions (positive vs. negative) against the actual labels. Table 4 summarizes model performance metrics on the Wildtracks test set, comparing results after training solely on the Saint George’s Caye dataset and after fine-tuning on the Wildtracks dataset, as well as performance of both models on their respective validation sets.

**Table 3:** Confusion matrix for the 2022 Wildtracks test set showing the number of TP (true positives) and FN (false negatives).

Predicted/Actual	Positive (1)	Negative (0)
Predicted Positive (1)	TP = 356	FP = 26
Predicted Negative (0)	FN = 7	TN = 21,375

**Table 4:** Results on the 2022 wildtracks test set and the SGC and wildtracks validation sets for the model that was only trained on the St. George’s Caye data (Stage 1) and the model after fine-tuning on the Wildtracks train set (Stage 2).

Model+dataset	Call F1 score	Precision	Recall	Accuracy	AUC-PR
2 (Stage 1) SGC val set	68.4%	73.6%	63.9%	99.4%	66.6%
2 (Stage 2) Wildtracks val set	90.1%	84.7%	96.2%	99.7%	97.1%
2 (Stage 1) test set	15.2%	8.4%	85.0%	62.9%	43.3%
2 (Stage 2) test set	95.6%	93.2%	98.1%	99.9%	98.8%

### Experiment 3: Generalization Test – Placencia

**Fig 4:**
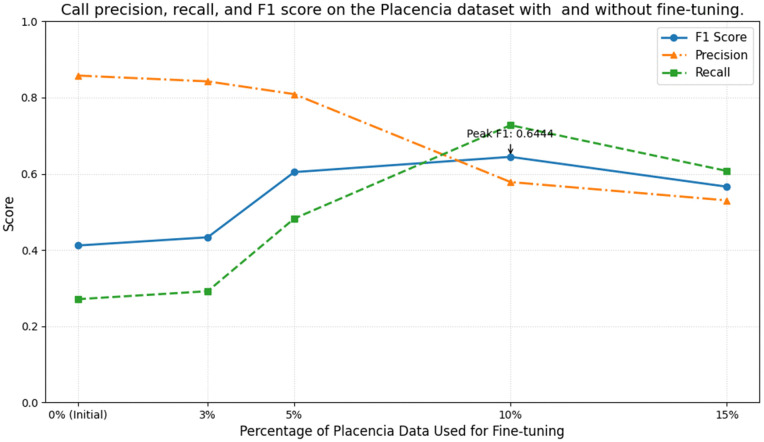
Call precision, recall, and F1 score on the Placencia dataset with and without fine-tuning.

**Fig 5:**
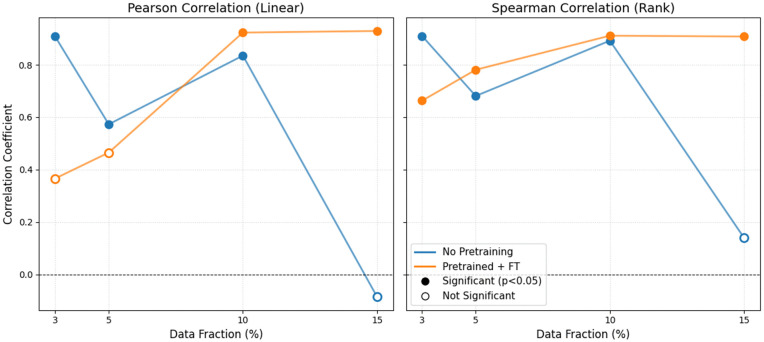
Effect of Data Fraction on Pearson and Spearman Correlation in Placencia: Fine-tuning (orange) vs. direct training without pre-training (blue). Significant values (p-value<0.05) marked with filled dot.

Following are the results of the Placencia generalization test before and after fine-tuning on small subsets of annotated data (Fig 4), as well as a comparison between fine-tuning our trained model vs. training a model directly on the small subsets of Placencia data, via correlation to the ground truth annotations (Fig 5).

**Fig 6:**
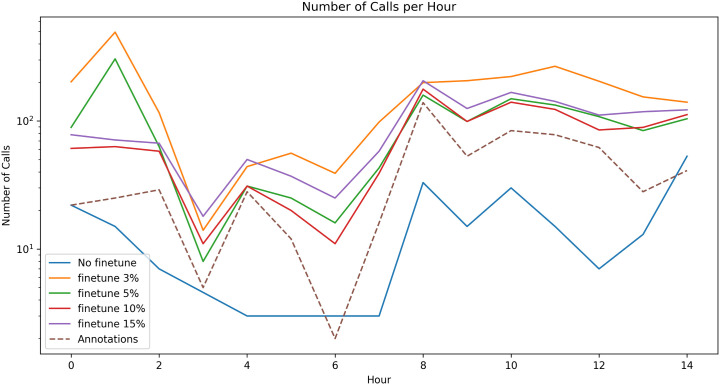
Number of identified calls per hour in the Placencia test recordings with the pre-trained model prediction (solid lines) and in the ground truth annotations (dashed line) with no fine-tuning and with fine-tuning on 3% to 15% of the Placencia set.

**Fig 7:**
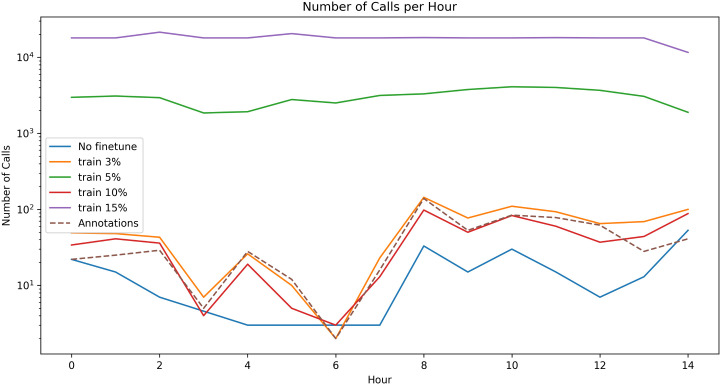
Number of identified calls per hour in the Placencia test recordings with model prediction (solid lines) and in the ground truth annotations (dashed line) with the pre-trained model and with models that were not pre-trained on any manatee set, then trained on 3% to 15% of the Placencia set.

Fig 6 and Fig 7 show the vocalization occurrence per-hour as a practical tool for finding activity peaks in large recording corpora, even if the model has relatively low precision, and compares the profile of the occurrence graphs after fine-tuning the pretrained model on data from the domain or training a model without pretraining.

## Discussion

In this study, we presented a convolutional neural network pipeline designed specifically for the detection of Greater Caribbean manatee vocalizations. Our approach combined advanced signal processing techniques (e.g., spectrogram optimizations, filtering, different normalization techniques) with deep learning algorithms. The three experiments demonstrate the detector’s potential for automated, large-scale monitoring. In the comparative experiment, we see that the ResNet model with the Adam optimizer outperformed GoogLeNet and the SGD optimizer across all metrics, achieving higher F1 score, accuracy, precision, recall, and AUC-PR (area under the precision-recall curve). Its superior performance, with fewer false positives and misdetections, highlights its effectiveness for this application.

In the domain adaptation experiment on the Wildtracks test subset, the detector achieved 99.9% overall accuracy, 95.6% call-level F1, 93.2% precision, and 98.1% recall, significantly higher than the metrics with the model only trained on the St. George’s Caye dataset. The high metrics, especially AUC-PR, on the large wildtracks validation set also indicates there was no overfitting on the small Wildtracks training set These figures confirm that, when training and deployment domains match closely, the ResNet18-2D can detect vocalizations with very high confidence.

Fine-tuning the pretrained checkpoint on only 3–15% of annotated Placencia data yielded substantial recall gains, with improvements leveling off around the 10% mark. Larger fine-tuning sets (> 10%) began to over-fit background noise, producing a marginal decline in F1 on the held-out test set. Thus a small, targeted annotation effort captures most of the attainable performance without exhaustive labelling. The correlation analyses (Pearson and Spearman) indicate that the pre-trained model (initially trained on the datasets from St. George’s Caye and Wildtracks) effectively preserves relative temporal activity patterns even after minimal fine-tuning on the Placencia dataset. While raw detection metrics (F1) remain lower in this challenging acoustic environment, we observe consistently high correlation coefficients (≥0.9) for both measures with as little as 10% target domain data, highlighting that the model captures and preserves the relative trends in call activity. These findings underscore the robustness of the learned acoustic representations and their adaptability across related recording environments. Models trained on small subsets without pre-training displayed lower and more erratic correlations – sometimes failing to generalize at all – whereas the pre-trained models remained robust. This contrast highlights the value of pre-training: exposure to related Belize recordings equip the network with transferable acoustic features, while training solely on a small target set leaves it unable to model consistent temporal patterns.

The application of neural networks to study manatee vocalizations is relatively recent, with limited research available on this topic. However, our findings align with previous studies that have shown the potential of neural networks in detecting manatee vocalizations [14,37]. Our study extends this research by focusing specifically on the Greater Caribbean manatee and is expected to yield better results as it accounts for the various acoustic environments they inhabit. Potential differences in season and habitat types may influence detection and accuracy of neural networks. Captive environments, seagrass habitat, and coastal areas have different acoustic profiles (e.g., filtration, habitat, bathymetry), which influence sound transmission loss, attenuation, and frequency-dependent propagation. As a result, manatee vocalizations may propagate differently across these environments, affecting both the characteristics of recorded calls and their detectability by acoustic monitoring systems [38,39]. These environmental differences pose a challenge for neural networks trained on data from a single habitat, as models, particularly CNNs, perform best when the test data closely resembles the training data [40]. If seasonal or habitat-based variability, especially in background noise, is not well represented in the training set, the model may misclassify or fail to detect vocalizations, reducing overall robustness and generalization. As shown by Rycyk et al. [41], even when using data from relatively quiet and acoustically similar sites, detection performance improved when training data included samples from the specific test location. This highlights the importance of incorporating site-specific recordings, particularly from acoustically distinct environments, into the training dataset to account for soundscape variability and improve detection accuracy. Consistent with these earlier observations, our own fine-tuning experiment on the acoustically distinct Placencia site showed that adding just 10% of site-specific annotations was enough to raise the detector’s hourly call-count correlations to ≥ 0.9 and recover most of the recall lost in zero-shot transfer, confirming that even a modest infusion of local data can markedly restore model reliability in a new soundscape.

Future research could build upon our findings by further optimizing and refining the neural network model to improve its detection accuracy. This could involve exploring different architectures, such as incorporating attention mechanisms or incorporating unsupervised learning techniques to better capture the inherent structure in the data [42,43]. Additionally, integrating other sources of information, such as environmental or behavioral data, could provide valuable context to improve the model’s performance and ecological relevance.

The successful application of neural networks to detect Greater Caribbean manatee vocalizations in different habitats could also be used to explore other aspects of manatee behavior, such as the relationship between vocalizations and social dynamics or the impact of anthropogenic noise on their communication patterns. For example, individual manatees exhibit stable acoustic features in their vocalizations [44], which suggests the potential for identifying individuals in an aggregation using neural networks. This approach that has been successfully applied to recognize similar dolphin signature whistles [45]. Further, CNNs can effectively classify anthropogenic noise, weather related phenomena, and ambient sound from soundscapes [46], which could aid in distinguishing boat noise from manatee vocalizations in sensitive habitats. This capability would enable researchers and managers to identify critical habitats where boat traffic noise is most prevalent and disruptive, and how it may influence manatee presence or behavior.

## Conclusions

This study presents a deep-learning-based framework for automated detection of manatee vocalizations, demonstrating the potential for data-driven conservation strategies. Our ResNet model outperformed previous methods, achieving high detection accuracy while maintaining strong generalization across different acoustic environments. The ability to fine-tune the model with minimal annotated data highlights its scalability for new monitoring sites

Furthermore, this methodology could be extended to support conservation efforts for other sirenian species, such as the African manatees, and applied to other regions where acoustic monitoring technology is currently lacking [47]. Its flexibility makes it particularly valuable across the range of habitats where manatees are found, including freshwater, brackish, and saline environments, each with distinct acoustic conditions that can influence detection. Additionally, classifying specific call types can yield ecologically meaningful insights. For example, the presence of high-pitched squeaks, a stereotypical call produced by young calves [48], may indicate cow/calf pairs in the area. Similarly, the detection of chewing or feeding-associated sounds can help identify important foraging habitats, providing a deeper understanding of habitat use and informing efforts to protect the resources essential for manatee survival.
